# Generation of the *Ci1* Reporter Mouse Strain with Enhanced Fluorescence for Tissue Clearing Applications

**DOI:** 10.1007/s12264-025-01421-4

**Published:** 2025-06-01

**Authors:** Manyu Chen, Youqi Li, Juan Huang, Yilong Wang, Hu Zhao

**Affiliations:** 1 School of Basic Medical Sciences, Capital Medical University, Beijing, 100069 China; 2 Department of Neurology, Beijing Tiantan Hospital, Capital Medical University, Beijing, 100070 China; 3 Chinese Institute for Brain Research, Beijing, 102206 China

**Keywords:** Reporter mouse strain, MScarlet fluorescent protein, TdTomato

## Abstract

The advancement of tissue clearing technology has significantly propelled neuroscience research. Nevertheless, the fluorescent proteins used in traditional transgenic mouse strains were not specifically optimized for tissue clearing procedures, resulting in a substantial decrease in fluorescent intensity after clearing. In this study, we developed the *Ci1* reporter mouse strain (where Ci stands for the Chinese Institute for Brain Research, CIBR) based on the bright red fluorescent protein mScarlet. The *Ci1* reporter exhibits no fluorescence leakage in various organs or tissue types and can be readily crossed with multiple tissue-specific Cre lines. Compared to the *Ai14* mouse strain, the *Ci1* reporter strain demonstrates lower non-specific leakage, stronger fluorescence intensity in different tissues, and better preservation of fluorescence following tissue clearing treatment. The creation of the *Ci1* reporter provides a more effective tool for both neuroscience and other biomedical research applications.

**Supplementary Information:**

The online version contains supplementary material available at 10.1007/s12264-025-01421-4.

## Introduction

Recent advances in tissue clearing technologies have revolutionized the 3D morphological analysis of biological tissues. These methods share common chemical principles of lipid removal and protein preservation through chemical treatment [1]. However, the chemical diversity required to clear heterogeneous tissues (e.g., bone *vs* neural tissue) risks fluorescent signal degradation, particularly for endogenous fluorescent proteins like tdTomato and GFP [2–4]. This challenge is amplified in peripheral nerve studies, where long-range axonal projections traverse multiple tissue types with distinct clearing requirements.

Given this situation, there is a need for a new type of reporter mouse model that can withstand harsher and more aggressive organic solvent treatment, with stable and strong fluorescent signals, minimal fluorescent leakage, and suitability for tissue clearing technologies based on organic solvents, to facilitate the morphological observation of both the central and peripheral nervous systems.

There are already many types of reporter mouse strains available for neuroscience research. *Thy1-EGFP/YFP* transgenic mice are among the first mouse models for neuroscience imaging tools. Dozens of transgenic mice strains were generated to express four different fluorescent proteins (GFP, EGFP, YFP, CFP) driven by the transgenic *Thy1* promoter [5]. Different types of neurons were relatively sparsely labelled which then enabled the details of densely arranged neurons to be visualized.

Cre-dependent fluorescent reporter strains provide a better way to label different neural tissues. The *Rosa26* locus has been the most widely used safe harbor for the expression of exogenous genes. Genes inserted into this region have high expression efficiency without affecting the normal expression and function of other genes, and they are not restricted by cell type or developmental stage [6, 7]. Consequently, the *Ai* series of mice were developed, with the genes for ZsGreen and tdTomato (the brightest fluorescent proteins at the time) knocked into the Rosa26 safe harbor, resulting in the creation of widely used Cre-driven fluorescent reporter mice including *Ai6, Ai9, Ai14*, and *Ai47*. [8]. Later, the TIGRE allele was identified and was combined with the tTA-TRE system to enhance the expression efficiency of the fluorescent protein genes. This led to the development of TIGRE1.0 triple transgenic mice (Cre x tTA x reporter gene) and the TIGRE2.0 strain [9, 10]. However, excessive tTA may lead to the formation of fluorescent aggregates in cells or abnormal morphology of labeled cells, as well as abnormalities in embryo growth when crossed with certain Cre strains [10].

More recently, the *H11* safe harbor, located between the *Eif4enif1* and *Drg1* genes on mouse chromosome 11, has provided a new safe harbor for expressing exogenous genes [11]. This allows the recombinant gene to be controlled by an independent promoter, which has been effectively validated in mice and other animal models [12–14]. In addition, new types of red fluorescent proteins with higher fluorescence intensity than tdTomato have been reported. Among many red fluorescent proteins, the *mScarlet* [15] red fluorescent protein has been effectively validated in Antarctic marine bacteria, *Caenorhabditis elegans*, zebrafish embryos, and human-induced pluripotent stem cells [16–19]. It offers higher brightness and maturation speed, stable fluorescence intensity, longer fluorescence lifetime, lower biological toxicity, and higher biological accumulation [15]. The longer wavelength also enhances imaging depth and contrast. In experiments with mouse models, adeno-associated viruses carrying the *mScarlet* gene have demonstrated superior fluorescence intensity and imaging performance [20]. However, transgenic reporter mice based on the *mScarle*t protein have not been available.

Here, we generated *Ci1* (Ci represents the CIBR) reporter mouse strain using *mScarlet* as the reporter fluorescent protein, to specifically label different tissues. Combined with tissue clearing technology, this new reporter strain provides a more efficient tool for analyzing neurons in both central and peripheral nervous systems.

## Materials and Methods

### Animal Models

Mice were purchased from the Jackson Lab (JAX, Bar Harbor, USA) with genotypes including Ai14 (Stock Number: 007914), Nav1.8-Cre (Stock Number: 036564), Mrgprd-CreER^T2^ (Stock Number: 031286), Wnt1-Cre (Stock Number: 022501), Vgat-Cre (Stock Number: 028862). All animal experiments were approved by the Institutional Animal Care and Use Committee of the Chinese Institute for Brain Research.

For tamoxifen treatment, tamoxifen (T5648, Sigma-Aldrich, Saint Louis, USA) was dissolved in corn oil (C8267, Sigma-Aldrich) at 20 mg/mL. The solution was kept at – 20 °C and delivered *via* intraperitoneal injection or oral gavage for postnatal treatments.

### Generation of the *Ci1* Mouse Model

We sequentially linked the triple copies of gene sequences of mScarlet fluorescent protein using P2 A and T2 A self-cleavage sites, and this overexpression was dependent on the presence of Cre. To ensure stable gene expression and to avoid disrupting the functions of original genes in the genome, the transgene sequence was inserted into the H11 site located on mouse chromosome 11. The sgRNA sequence was 5‘-GAACACTAGTGCACTTATCC-3’, and the synthesized template was obtained by PCR and purified using the Qiagen (Hilden, Germany) MinElute kit, carrying a T7 promoter. The sgRNA was synthesized using the Ambion MEGAshortscript kit (Thermo Fisher Scientific, Waltham, USA) and then purified using the MEGAclear kit (Thermo Fisher Scientific). Through PCR, the three copies of mScarlet, P2 A, and T2 A were spliced together and inserted into a vector that already carried homologous arms and CAG-LSL-MCS-WPRE-pA. The homologous vector, *in vitro,* synthesized Cas9 mRNA, and sgRNA were injected into the fertilized eggs of C57 BL/6 J mice. To genotype the offspring, these three primers were used F3-1: 5‘-GCTCATTAGATGCCATCATGCTCTC-3’; F3-2: 5‘-TAGTTGCCAGCCATCTGTTG-3’; R3: 5‘-TGGGTCTTCCACCTTTCTTCAGTTAG-3’. A 447-bp band was expected to be amplified by primers F3-1 and R3 in the WT allele, while a 548-bp amplicon from primers F3-2 and R3 was only expected in KI mice. We used the Vazyme (Nanjing, China) One Step Mouse Genotyping Kit-PD101 for PCR genotyping. The PCR protocol was as follows: 5 min of initial denaturation at 94 °C, followed by 35 cycles of 30 s of denaturation at 94 °C, 30 s of annealing at 55 °C, and 30 s of extension at 72 °C, and then a final extension at 72 °C for 7 min.

All experimental protocols complied with the guidelines of the IACUC of the Chinese Institute for Brain Research.

### AAV Injection

The virus AAV2/9-hsyn-Cre was provided by the Vector Engineering Core of CIBR (Beijing, China). According to the mouse brain atlas (second edition), the exact coordinates of the injection sites were determined by the distances from bregma in the mediolateral (ML), anteroposterior (AP), and dorsoventral (DV) directions. The brains were collected after perfusion with 4% paraformaldehyde. Stereotactic brain injections were made into the hippocampus (ML 2.1, DV 2.18, AP −1.6 mm) or the M1 cortical area.

### Sample Processing, Histology, and Immunofluorescence

Three weeks after viral injection, mice were sacrificed under anesthesia, and the heart was perfused with 1× phosphate-buffered saline (PBS) and fresh 4% paraformaldehyde (PFA) to completely remove blood. The brains were post-fixed in fresh 4% PFA overnight at 4 °C, washed three times with 1 × PBS, dehydrated in 60% sucrose at 4 °C overnight, and then embedded in OCT compound the next day before preparing cryosections. All frozen samples were sectioned at 20 μm. The sections were stained with DAPI for nuclear labeling, and then imaged using the Olympus (Tokyo, Japan) VS120 digital slide workstation and the Nikon (Tokyo, Japan) AX R with an NSPARC confocal microscope.

### Cell Counting and Quantification

Imaris 9.0.1 was used to count the illuminated neuronal cell bodies, and to measure fluorescence intensity. GraphPad Prism 8.0.2 was used to draw bar charts. All statistical analyses (Student’s *t* tests) were applied using GraphPad Prism 8.0.2, and all quantification data are presented as the mean ± SEM. ns: *P* > 0.05, *P ≤ 0.05, **P ≤ 0.01, ***P ≤ 0.001, ****P ≤ 0.0001.

### Tamoxifen Injection

Tamoxifen was dissolved in corn oil or DMSO at 20 mg/mL as the working solution. Mice were injected intraperitoneally at a dosage of 0.15 mg/g body weight per day for 1–3 days.

### Measurement of Fluorescence Changes after PEGSOS Treatment

We used dorsal root ganglia sections to measure the fluorescence changes following the steps of the PEGSOS tissue-clearing method. Sample slides were immersed in the solutions for each step on a 37 ℃ shaking bed for one day (80 r/min), and imaged using the Nikon AX R with an NSPARC confocal microscope. The same sample was imaged under the same conditions throughout the process. We used Cellpose 1.0 (https://www.cellpose.org/) and Fiji to statistically analyze the fluorescence intensity of neuronal cell bodies and used GraphPad 8.0.2 to draw line charts. All quantification data are presented as the mean ± SEM. ns: *P* > 0.05, *P ≤ 0.05, **P ≤ 0.01, ***P ≤ 0.001, ****P ≤ 0.0001.

### TESOS Tissue Clearing and Deep Imaging.

The forepaw of adult *Nav1.8-Cre;Ci1* mice (6–8 weeks old) was imaged using the TEOSOS method as previously described. In brief, the samples were fixed with 4% PFA at 4 °C for 24 h. Subsequently, decalcification was carried out in 20% EDTA (pH 7.0) at 37 °C on a shaker for 4 days. After decalcification, the samples were washed with distilled water for at least 30 min to remove excess EDTA. Next, they were decolorized with Quadrol solution for two days at 37 °C on a shaker. Then, the samples were placed in gradient tB delipidation solutions for 1–2 days, followed by tB-Q dehydration solution for 2 days. Finally, they were immersed in BB-BED medium in a 37 °C shaker for at least one day until transparency was achieved.

Once sample transparency was achieved, transparent embedding could be performed. The samples were irradiated on ice with a high-power UV curing light (Thorlabs, Newton, USA, CS20 K2) with a typical power of 50 mW/cm^2^ for a cleared mouse brain sample. After UV curing, the sample block was preserved in a 50-mL tube containing 3 mL – 5 mL of BB-BED medium.

The embedded samples were then mounted on a MagMount setup and imaged with a 40×/1.3 NA objective on a Nikon AX confocal microscope in combination with a microtome. The voxel size was 0.4 µm × 0.4 µm × 1.5 µm. The sample was imaged to a depth of 350 µm each time and then a microtome was used to remove the surface 300 µm. Eighteen sectioning procedures were performed. The image data was processed and stitched with ImageJ and displayed with Imaris 10.0.

## Results

### Transgenic Construction Strategy for the mScarlet-based Fluorescent Reporter Mouse Strain *Ci1*

**Fig. 1:**
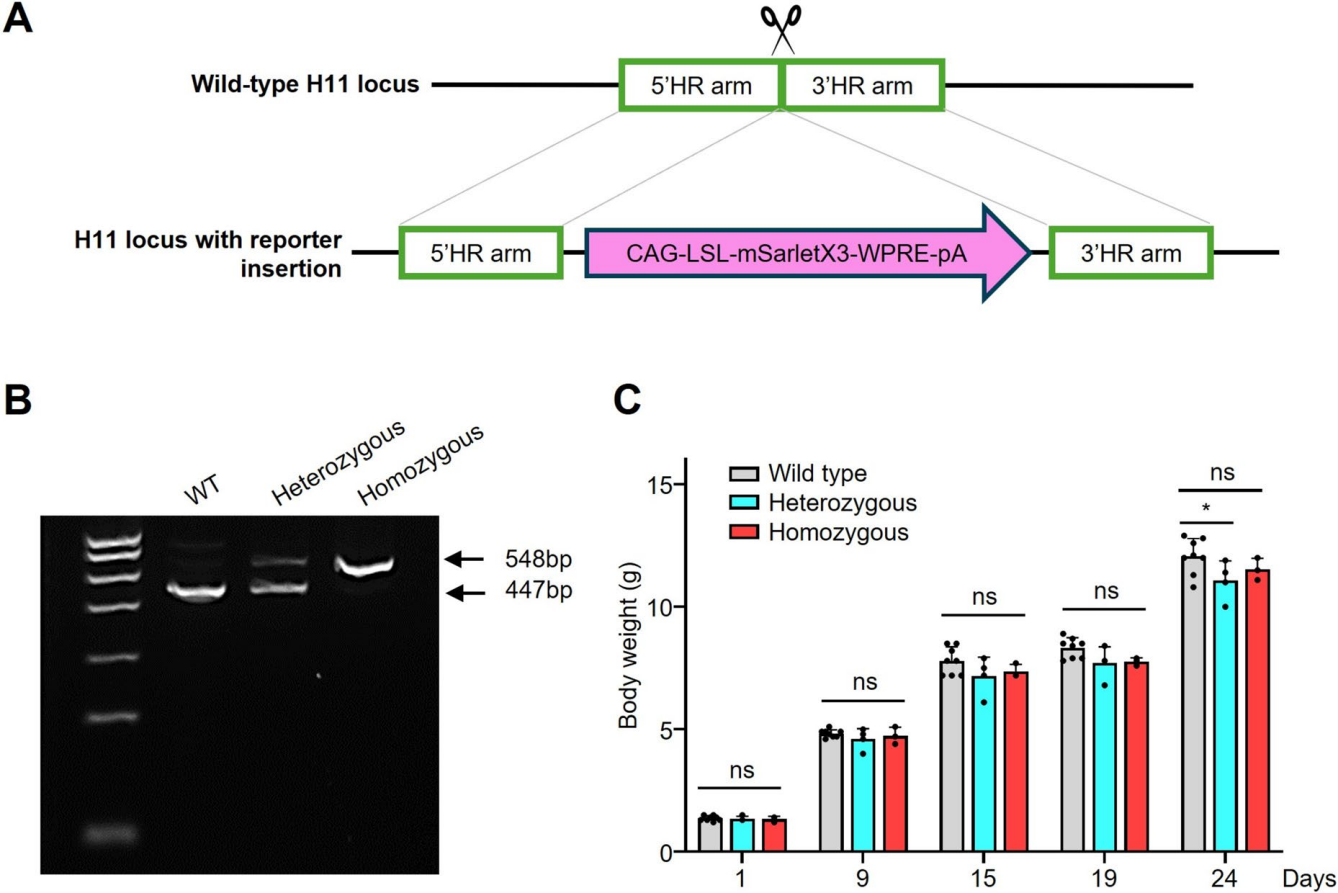
Generation of the Ci1 reporter mouse strain. **A** Schematic of the gene-targeting strategy for inserting the 3×mScarlet reporter cassette into the H11 locus. **B** The agarose gel electrophoresis results of the genes from heterozygous and homozygous mice of both the wild-type and *Ci1* lines after probe PCR amplification. **C** Weight gain of the *Ci1* strain and C57/BL6 mice at different ages. ns: *P* > 0.05; *P < 0.05.

Here, we generated a new mouse model: *Ci1*. To ensure stable expression of mScarlet without disrupting native genomic functions, three copies of the fluorescent protein mScarlet were tandemly linked *via* P2 A and T2 A self-cleaving sites and then inserted as a transgene into the H11 locus on chromosome 11 of C57BL/6 J mice through targeted integration (Fig. 1A, B). Long-term growth and body weight monitoring of stably-inheriting offspring confirmed that the normal survival and healthy development of this reporter mouse remained unaffected (Fig. 1C).

### No non-specific Fluorescence Leakage is Detected in Various Tissues of *Ci1* Homozygous Mice

**Fig. 2:**
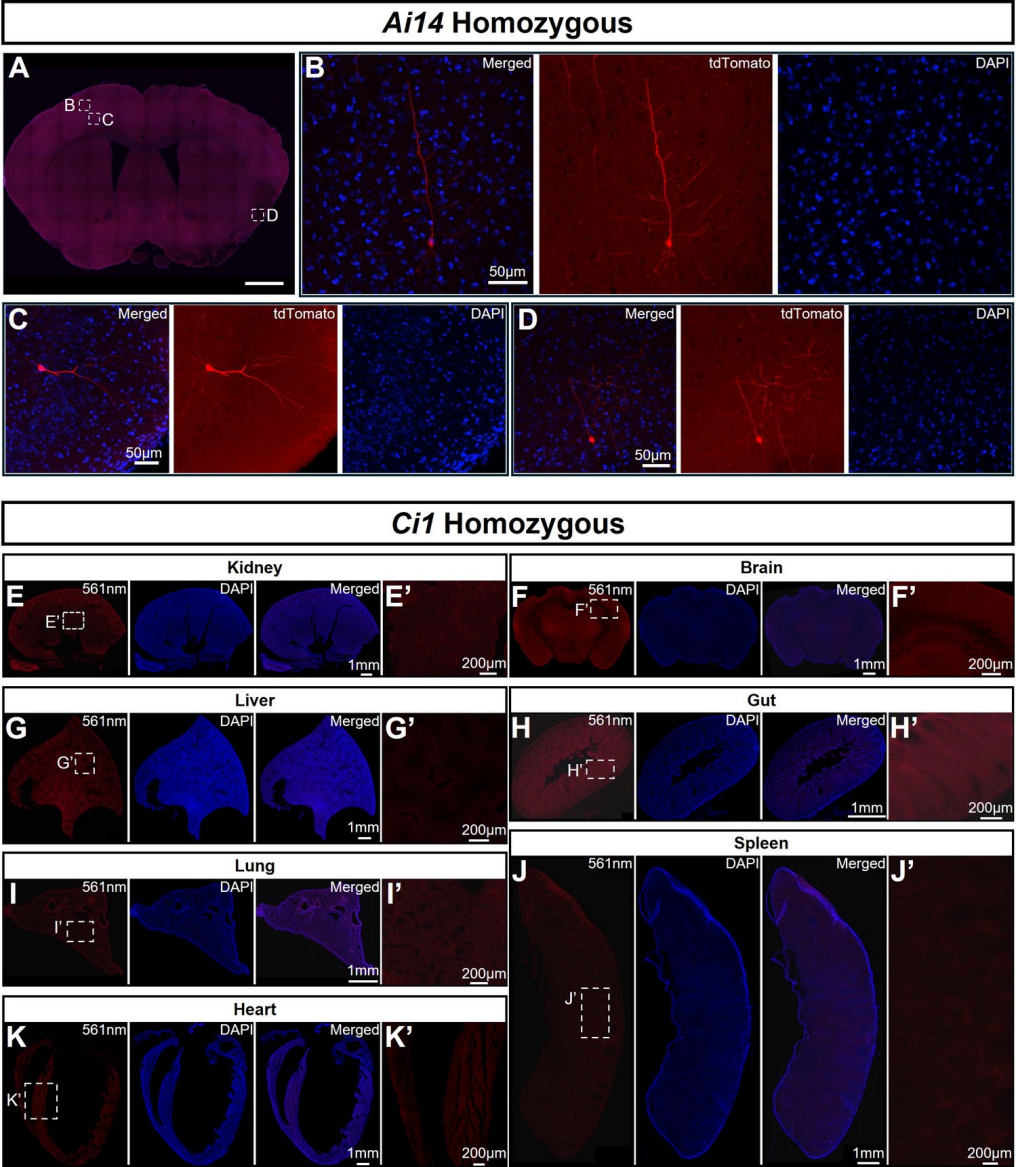
No non-specific fluorescence leakage is detected in various organs of *Ci1* homozygous mice. Panel **A** shows non-specific fluorescence leakage in the brain of 3-month-old homozygous *Ai14* mice. Panels **B**–**D** are magnified views. **E**-**K** Frozen sections of kidney (**E**), brain (**F**), liver (**G**), gut (**H**), lung (**I**), spleen (**J**), and heart (**K**) from 3-month-old homozygous *Ci1* mic exhibit no non-specific fluorescence leakage. Panels **E’**–**J’** are magnified views of their corresponding panels **E**–**J**. Cell nuclei are stained with DAPI.

The advantage of the Rosa26 locus lies in its high expression efficiency for transgenes inserted at this safe harbor site. However, this also brings certain side effects. In *Ai*-series mice generated using Rosa26 as the safe harbor site, non-specific fluorescence leakage has been observed. We specifically investigated this phenomenon in various organs of adult *Ai14* and adult *Ci1* homozygous mice. In adult *Ai14* homozygous mice (lacking Cre/CreER^T2^ driver elements) as controls, while low-level fluorescence leakage was detected in different locations of the brain, which revealed the morphology of neurons (Fig. 2A–D). We also evaluated the *Ai140* reporter strain. In adult *Ai140* homozygous mice, extensive non-specific leakage was detected at various locations in the brain (Fig. S1A–G). The brain of a 6-month Ai47 mouse was examined, and there was no non-specific fluorescence leakage (Fig. S1H, I). In contrast, in adult *Ci1* reporter mice, frozen sections showed that no fluorescence leakage occurred in various organs including the kidney, liver, lung, heart, brain, intestine, and spleen (Fig. 2E-J).

### *Ci1* Mice Show A Stronger Fluorescence Signal Intensity than Ai14 in AAV Labelling Experiments

**Fig. 3:**
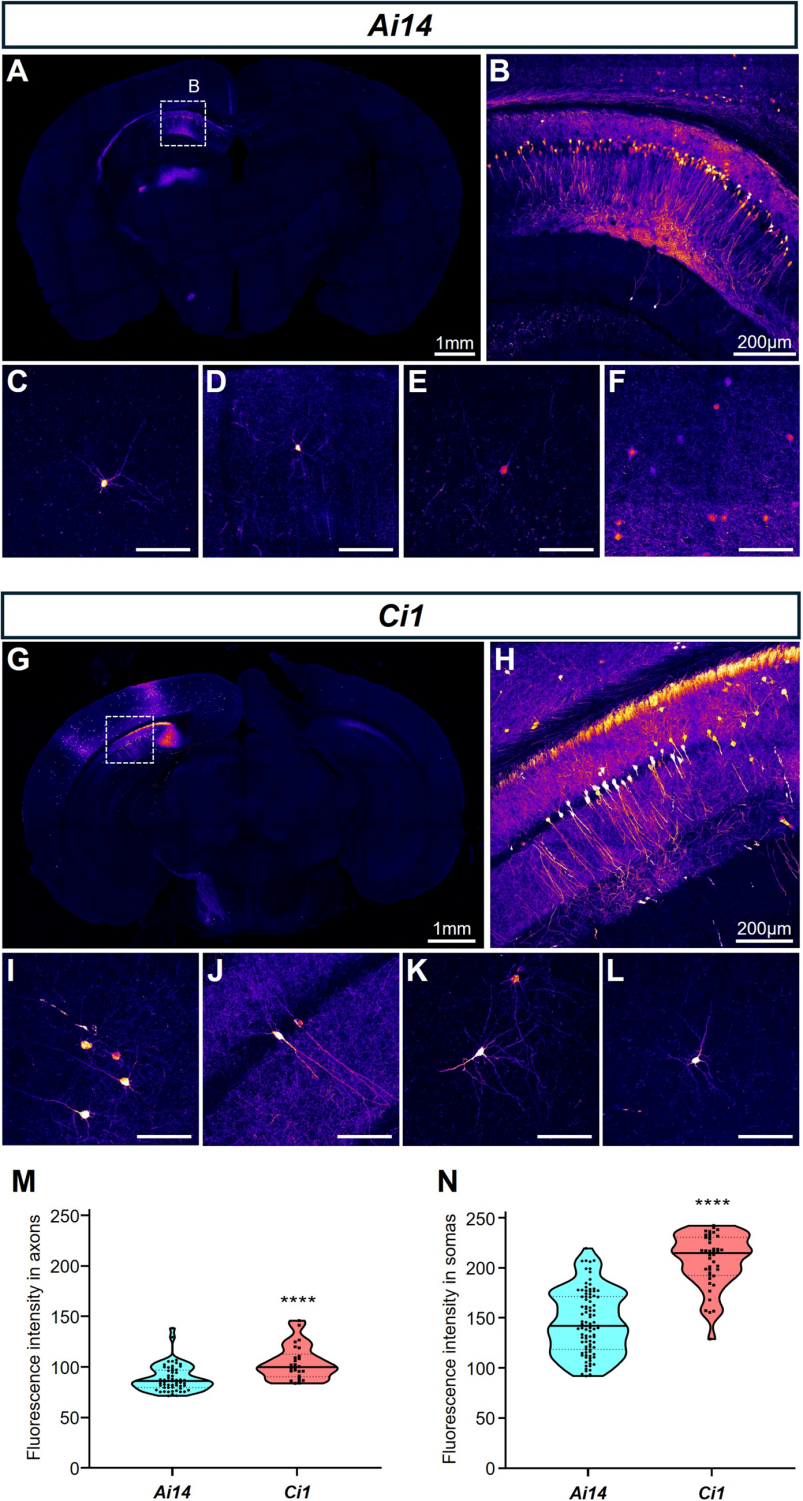
*Ci1* mice demonstrate highly efficient fluorescent labeling in AAV injection experiments in the brain.** A**–**F** and **G**–**L** Sections of the hippocampus location in *Ai14* (**A**–**F**) and *Ci1* (**G**–**L**) reporter mice after injection of the same dose of *AAV2/9-hSyn-Cre* at the same location, with individually labeled neurons magnified to show the structure (scale bars **in C**–**F** and **I**–**L**: 100 μm). Fluorescence intensity of the soma (**M**) and axon (**N**) of neurons expressing fluorescent proteins quantified using a *Student’s t-test*. ****P < 0.0001.

To test the performance of the *Ci1* reporter in AAV injection experiments to label neurons, we injected the same dosage and titter of *AAV2/9-hSyn-Cre* virus into the secondary motor cortex (M2) and the underlying hippocampus regions of homozygous adult *Ci1* mice or *Ai14* mice. Neurons in these two regions were both clearly labelled in both strains (Fig. 3A–L). Quantification showed that fluorescence intensity in both the somas and axons of *Ci1* mice was brighter than the tdTomato signal of *Ai14* mice (Fig. 3M, N).

### *Ci1 *Reporter Mice Label Peripheral Nerves Better than Ai14

**Fig. 4:**
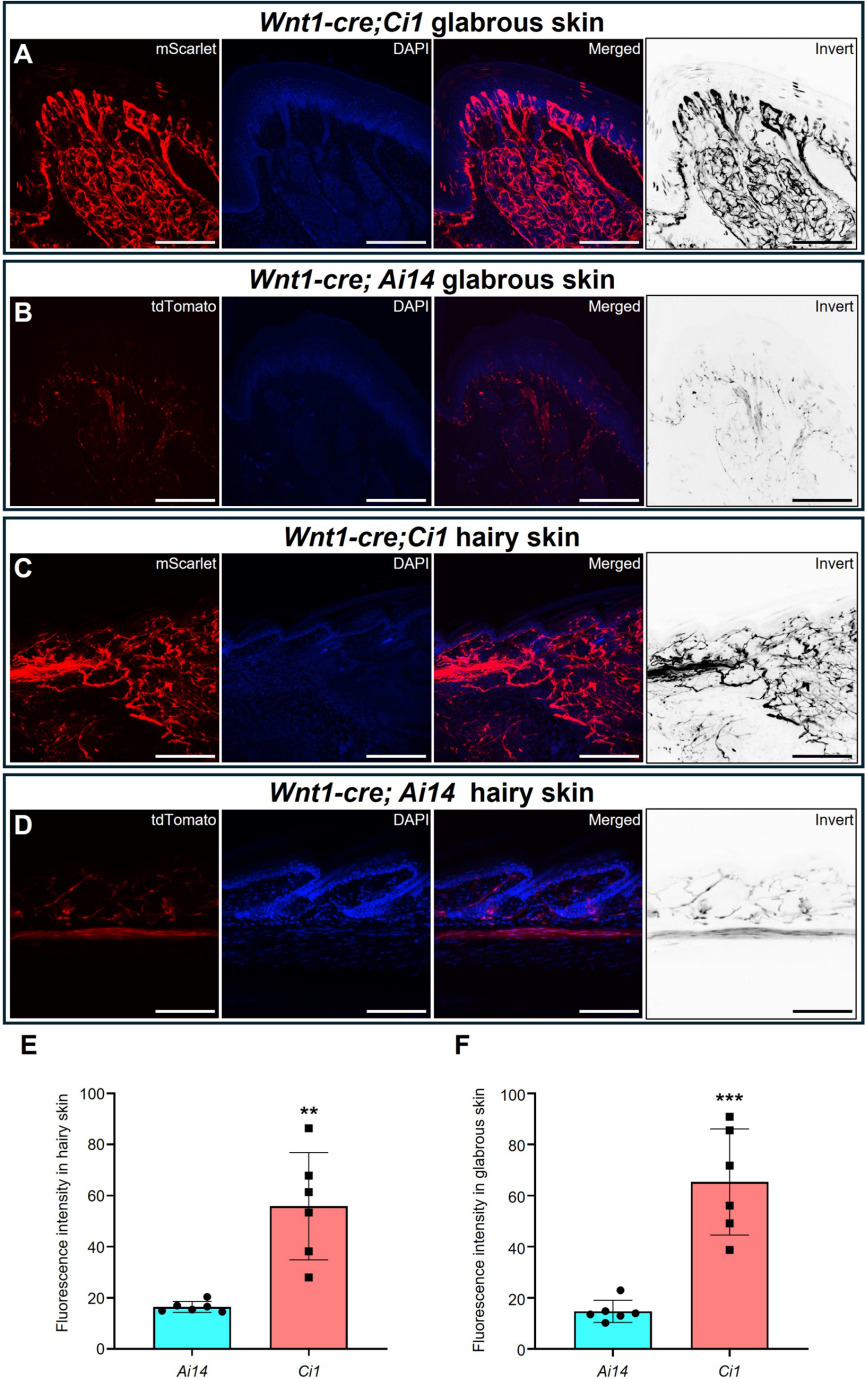
*Ci1* exhibits higher efficiency than Ai14 in labeling peripheral nerve endings beneath the skin. **A**, **C** The expression patterns in the skin of adult *Wnt1-Cre;Ci1* mice; **A**, glabrous skin; **C,** hairy skin. Scale bars are 50 μm. **B** and **D** illustrate the expression patterns in the skin of adult *Wnt1-Cre;Ai14* mice, with panel **B** depicting glabrous skin and panel **D** depicting hairy skin. Scale bars, 50 μm. **E**, **F** Fluorescence intensities in glabrous skin (**E**) and hairy skin (**F**) (n = 6). **P < 0.01, **P < 0.001.

In the peripheral nervous system, the cell bodies of sensory neurons are located within the craniofacial and dorsal root ganglions (DRG). Their axons have long projection distances, and fine and dense branches, and the areas they innervate are extensive and scattered. *Wnt1-Cre* labels the neural crest cells and therefore labels all peripheral neurons and glial cells [21]. After crossing with *Ci1* or *Ai14* the axons located under the glabrous skin of the forepaw digit tips were clearly labelled (Fig. 4A, B). The free nerve endings under hairy skin of the forepaw digit tips were also labelled in both *Wnt1-Cre;Ai14 flox/+* and *Wnt1-Cre;Ci1 flox/+* strains (Fig. 4C, D). Quantitation indicated that the fluorescence intensity of mScarlet was 2–3-fold stronger than those of tdTomato under both hairy skin and glabrous skin (Fig. 4E, F).

### *Ci1 *Reporter Mice can be Crossed with Various Tissue-specific Cre Transgenic Mice

**Fig. 5:**
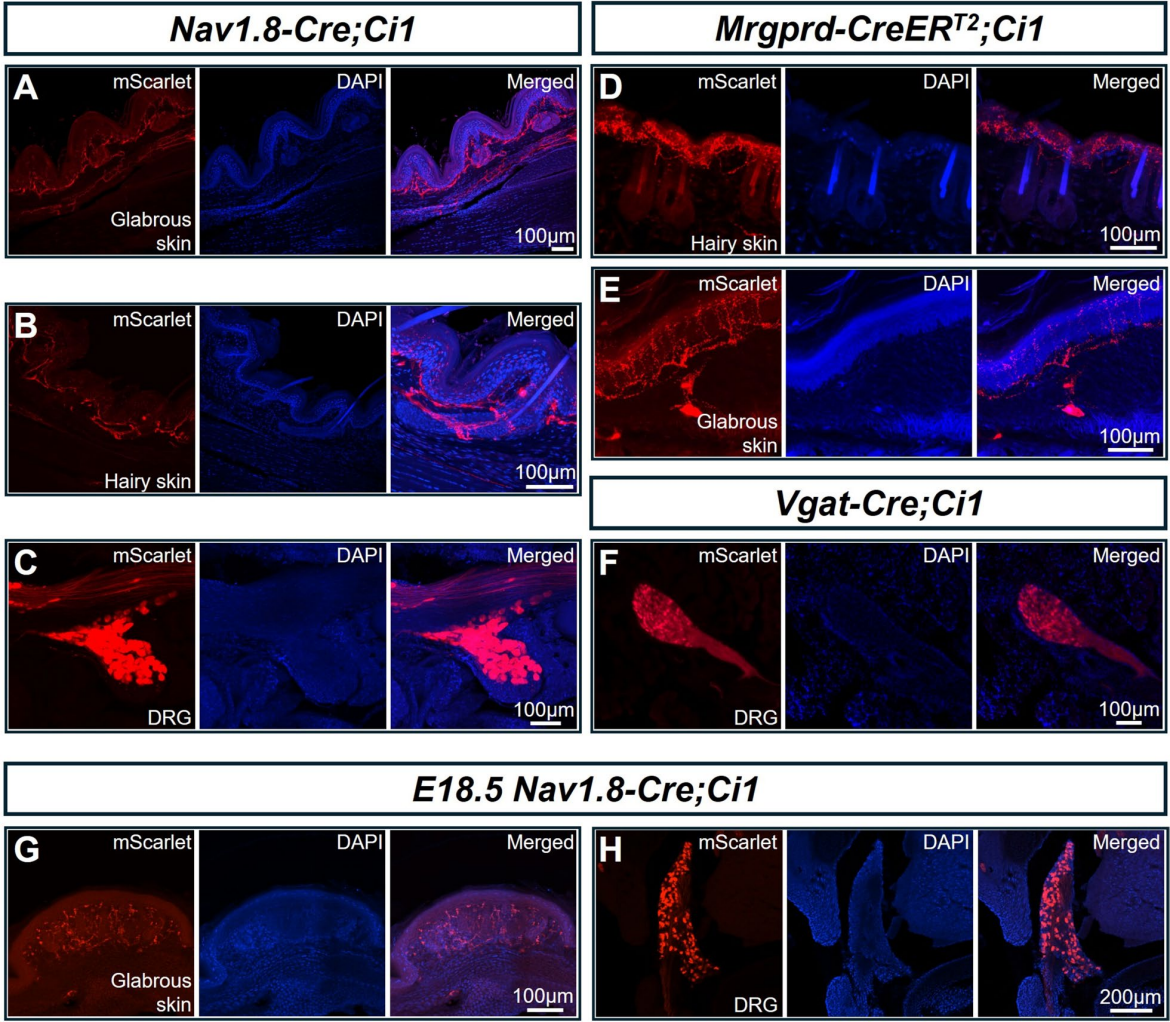
*Ci1* can be crossed with various neuron-specific Cre strains to specifically label peripheral nerves. **A-C** Fluorescence expression in glabrous skin, hairy skin, and the DRG in the cervical vertebrae of *Nav1.8-Cre;Ci1* mice. **D, E** Fluorescence signal in the glabrous skin and hairy skin of adult *Mrgprd-CreER*^*T2*^*;Ci1* mice one month following tamoxifen induction. **F** Fluorescent signal in the cervical DRG of *Vgat-Cre;Ci1* mice. **G**, **H** Fluorescence signal in the glabrous skin and DRG of *Nav1.8-CreER*^*T2*^*;Ci1* mice at E18.5.

*Ai140* strains were previously reported to undergo embryonic lethality when crossed with certain tissue-specific Cre strains [10]. In our experiments, attempts to breed *Ai140* mice with Wnt1-Cre or Nav1.8-Cre mice were similarly unsuccessful, likely due to embryonic lethality (data not shown). To determine whether *Ci1* mice are compatible with other Cre lines besides *Wnt1-Cre*, we crossed *Ci1* mice with *Nav1.8-Cre, Vgat-Cre*, and *MrgprD-CreER*^*T2*^ strains. Healthy offspring were obtained and successfully reared to adulthood for all three Cre/CreER^T2^ lines. In *Nav1.8-Cre;Ci1* mice, nociceptive axons in glabrous, hairy skin and DRG were clearly visualized (Fig. 5A–C). *MrgprD-CreER*^*T2*^*;Ci1* mice also reached adulthood, with strong fluorescent signals in both hairy and glabrous skin one month after tamoxifen induction (Fig. 5D–E). Similarly, *Vgat-Cre;Ci1* mice developed normally to adulthood, exhibiting robust fluorescent signals in DRG neurons (Fig. 5F). To demonstrate the usefulness of *Ci1* in embryonic studies, we examined *Nav1.8-Cre;Ci1* mice at E18.5. Both the somas in the DRG and the terminals of the neurons under the glabrous skin showed bright fluorescent signals (Fig. 5G, H).

### Fluorescence Intensity of* Ci1 *Reporter Remains Stable during Solvent-Based Tissue-clearing Treatment

**Fig. 6:**
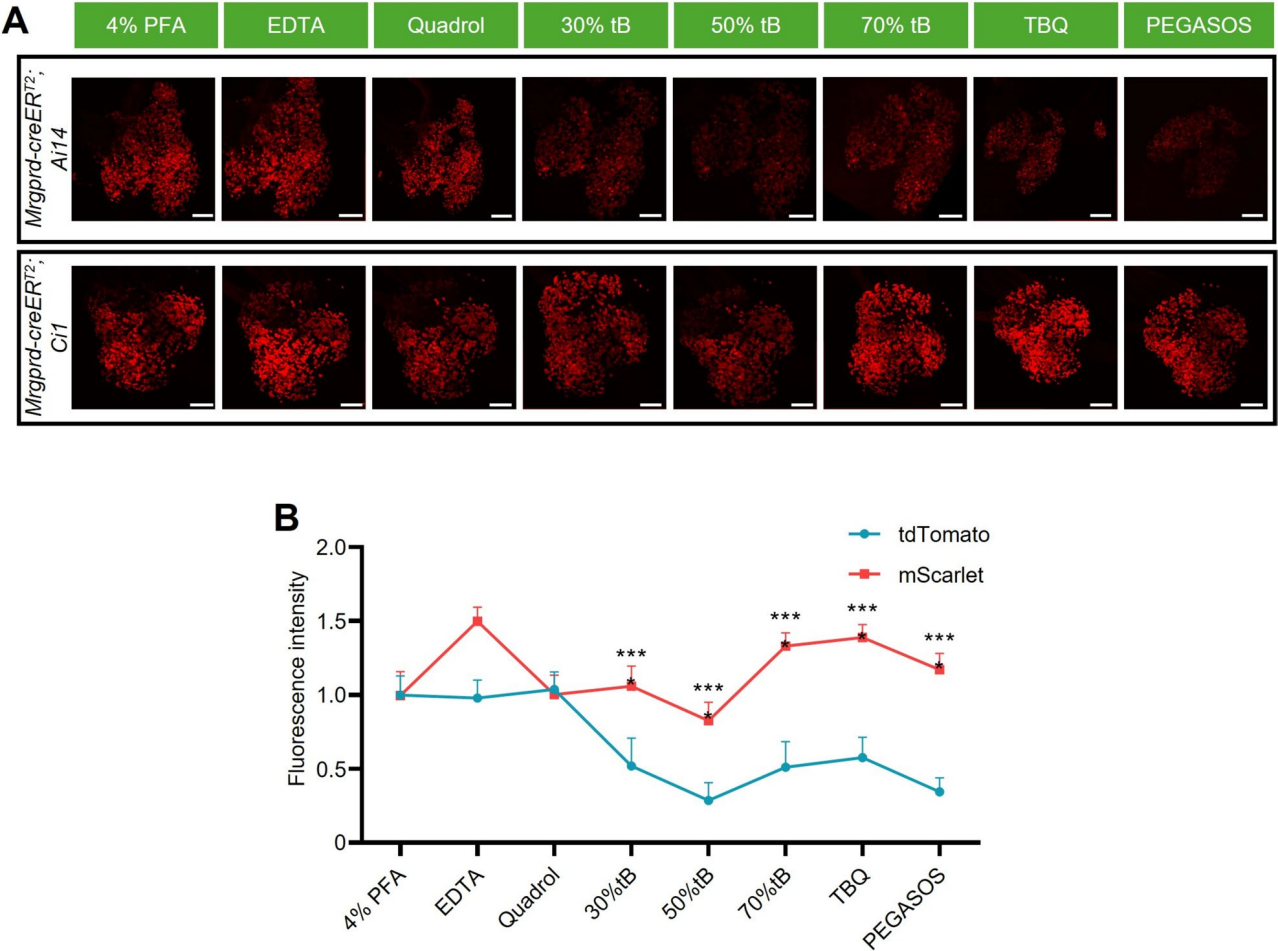
*Ci1* presents better fluorescence stability than *Ai14* during PEGASOS tissue-clearing treatment. **A** Impact of treatment steps in the PEGASOS method on fluorescence intensity of *Ai14* and *Ci1* samples. Scale bars: 200 μm. **B** Quantification of fluorescence intensity changes. ***P < 0.001.

Tissue-clearing techniques are of great importance for neural science research. However, these clearing techniques have high requirements for the intensity and stability of labeled fluorescence. To test the fluorescence stability of *Ci1* mice during the tissue-clearing process, we applied the PEGASOS (Polyethylene glycol (PEG)‐associated solvent system) [22] clearing method to treat the DRG of *Mrgprd-CreER*^*T2*^*;Ai14* or *Mrgprd-CreER*^*T2*^*;Ci1* mice. Fluorescence images were acquired after each treatment step using comparable parameters (Fig. 6A). During the treatment process, the fluorescence intensity of tdTomato gradually declined, and was finally maintained at ~40% of the initial intensity (Fig. 6B). In contrast, the fluorescence intensity of mScarlet remained stable after all the treatment steps. After clearing, the fluorescence intensity was at almost the same level as the initial intensity (Fig. 6B). This indicated that mScarlet protein is significantly more stable than tdTomato during solvent-based tissue-clearing treatment.

### The* Ci1 *Reporter Enables Sub-micron Resolution Deep Imaging of the Forepaw in Combination with the TESOS Method

**Fig. 7:**
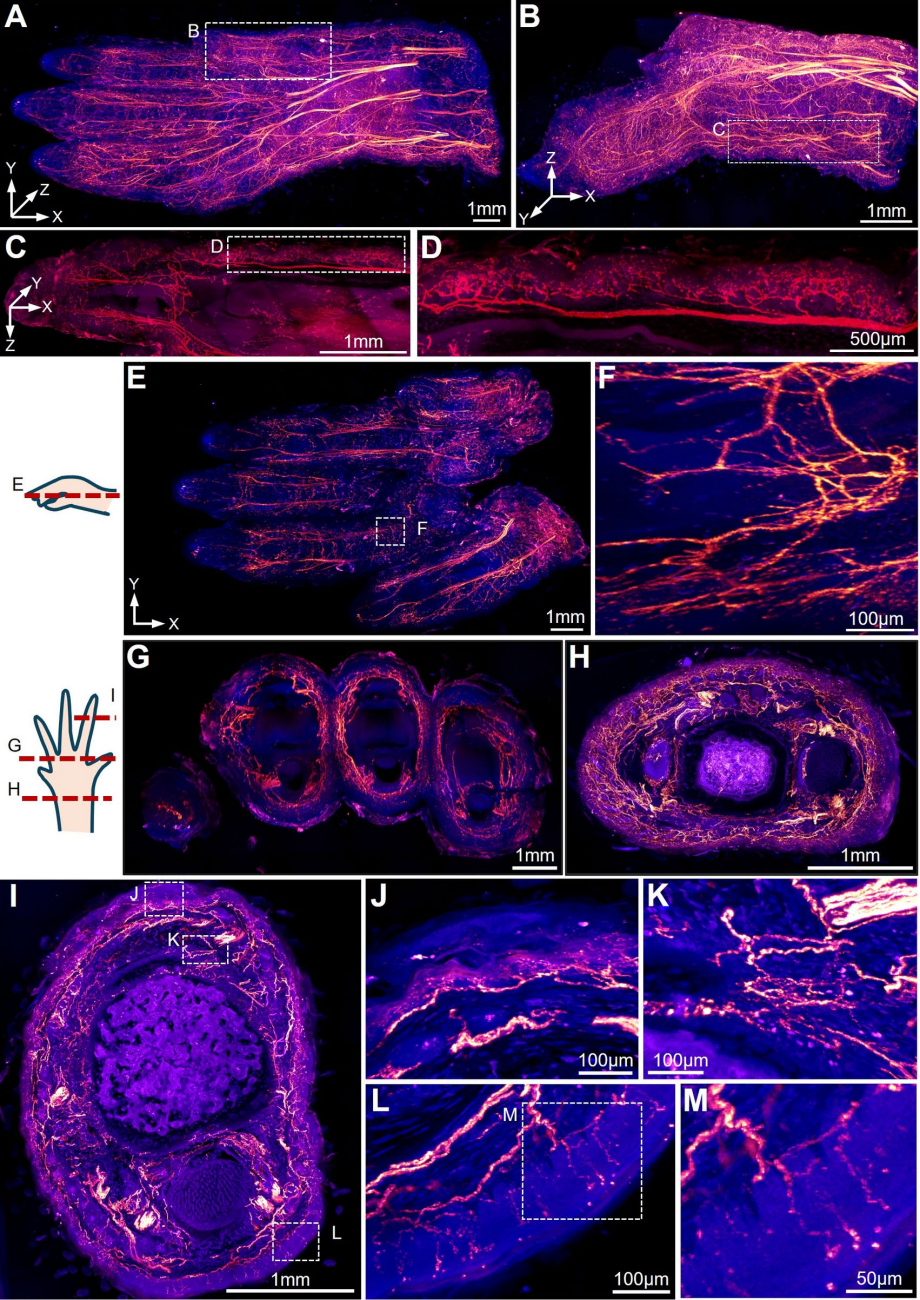
Three-dimensional imaging of an adult *Nav1.8-Cre;Ci1* mouse forepaw using the TESOS tissue-clearing method. The images were acquired with a 40×/1.3 NA objective on a confocal microscope. The sample size is 4.59 mm × 2.35 mm × 13.6 mm, with a voxel size of 0.4 µm × 0.4 µm × 1.5 µm. **A** A 3-D rendering of a forepaw sample; **B** the dotted box is enlarged to display the digit, **C** nerve axons beneath the skin, and **D** the terminal arbors within the dermis. **E** Horizontal and **G**–**I** cross optical sections. Boxed region in (**E**) is enlarged in **F**. Boxed regions in (**I**) are enlarged in the right panels **J**–**L** to show terminal axons under the skin. Boxed region in **L** is enlarged in **M**. The red signal represents mScarlet, and the blue signal indicates autofluorescence.

High-resolution deep imaging of peripheral nerves has been a challenge, mostly due to the strong autofluorescence derived from hair, skin, and muscles. Deep imaging in combination with Tissue-clearing methods has been achieved by using Thy1-EGFP or Thy1-YFP mouse strains due to their strong EGFP/YFP expression in neuronal tissue. Quenching after tissue-clearing treatment made it difficult for the traditional *Ai14* reporter to be utilized with multiple Cre lines to visualize peripheral nerve axons using Tissue-clearing approaches. Here, we generated *Nav1.8-Cre;Ci1* mice and harvested the forepaw from adult mice aged 6–8 weeks. Samples were processed following the TESOS method (Transparent Embedding Solvent System) [23]. The transparently embedded forepaw samples were mounted on MagMount and imaged with a 40×/1.3 NA objective on a confocal microscope equipped with a microtome. We were able to image the forepaw sample (4.59 mm × 2.35 mm × 13.6 mm) with a sub-micron resolution of 0.4 µm × 0.4 µm × 1.5 µm (Fig. 7A–M). All the nociceptive axons within the forepaw were clearly visualized. Consistent resolution was achieved throughout the entire sample (Fig. 7A–M).

## Discussion

In this study, we developed a novel fluorescent reporter mouse strain (designated *Ci1*) exhibiting superior fluorescence intensity and robust compatibility with solvent-based tissue-clearing methods compared with traditional reporter strains.

The H11 gene locus, instead of ROSA26 and TIGRE loci, was strategically selected as the recombination site for fluorescent protein integration and effectively eliminated nonspecific signal leakage in the absence of Cre recombinase. A previous study found that the ROSA26 locus has a higher transgene expression efficiency than the H11 locus [24]. This may explain why some *Ai* series Cre-dependent reporter mouse lines show non-specific fluorescence leakage.

The selection of mScarlet over conventional tdTomato was based on several critical biophysical advantages. For the first, despite tdTomato's 16–20% higher baseline brightness [15, 25], mScarlet's monomeric structure and lower molecular weight (26.4 kDa *vs* tdTomato's 54.2 kDa dimeric configuration) enables higher cytoplasmic expression levels and better transportation efficiency in the axon, which leads to higher fluorescent intensity in tissue. Second, mScarlet presents better photostability than tdTomato (t₁/₂ = 277 s vs. 70 s under 561-nm illumination) [15], which is extremely useful for long duration imaging. Finally, we showed mScarlet also possesses much stronger chemical stability during tissue-clearing than tdTomato.

Our analysis was based on a solvent-based tissue-clearing method. Solvent-based approaches (e.g., PEGASOS, iDISCO) are chemically harsher than aqueous methods (CLARITY, CUBIC) due to their use of organic solvents like dichloromethane, tert-butanol and ethanol dehydration, which induce stronger protein denaturation and fluorescence quenching [22, 26–28]. The robust performance of mScarlet in *Ci1* mice under these extreme conditions suggests even better compatibility with milder aqueous methods. All tissue-clearing methods subject proteins to denaturing conditions, including extreme pH, ionic strength changes, and prolonged chemical exposure [29–31]. Solvent-based methods combine multiple stressors, effectively serving as a reliable"stress test"for fluorescent probes. While specific protocols may require optimization (e.g., adjusting clearing duration for thicker organs), *Ci1*'s core advantages, especially its chemical stability, address universal challenges in 3D tissue imaging. These features make *Ci1* a versatile reporter system compatible with various clearing methodologies.

The unique features of the *Ci1* reporter make it particularly suitable for labeling peripheral nervous tissue. In peripheral tissue imaging, strong autofluorescence from bone, keratinized epithelium, hair, muscle, and blood creates major challenges for 3D tissue-clearing [29]. Traditional fluorescent proteins (e.g., GFP, YFP, tdTomato) often show severe fluorescence loss after clearing treatments, causing weak nerve signals to be masked by tissue autofluorescence. Antibody staining was commonly performed to boost signal strength in many peripheral nerve studies based on tissue-clearing approaches [32]. Another challenge comes from the long distances between neuron somas and endings. The somas of peripheral nerves are located in cranial or dorsal root ganglia, while their sensory or motor endings in the trunk and limbs are far away. Fluorescent proteins synthesized in the soma must travel long distances to reach nerve endings, where their levels become much lower than in the cell bodies. The smaller size of mScarlet (26.4 kDa) compared to tdTomato (54.2 kDa) allows better protein production and transport along axons. This difference explains why the *Ci1* reporter labels nerve endings much better than *Ai14*. These improvements make *Ci1* a valuable tool for studying the peripheral nervous system.

While our current investigation focused on neural applications, the *Ci1* reporter can clearly be combined with other tissue-specific Cre strains for broader applications in diverse organs and tissue types.

## Supplementary Information

Below is the link to the electronic supplementary material.

Supplementary file1 (PDF 388 kb)
