# Supplementary material for: Generation of the Ci1 Reporter Mouse Strain with Enhanced Fluorescence for Tissue Clearing Applications
Source: Neurosci Bull. 2025 Jun 1;41(8):1317–28. doi: 10.1007/s12264-025-01421-4 (PMC12314181; doi:10.1007/s12264-025-01421-4)
Supplement: Supplementary file 1 — Supplementary file1 (PDF 388 kb) [file 12264_2025_1421_MOESM1_ESM.pdf]

Supplementary Materials

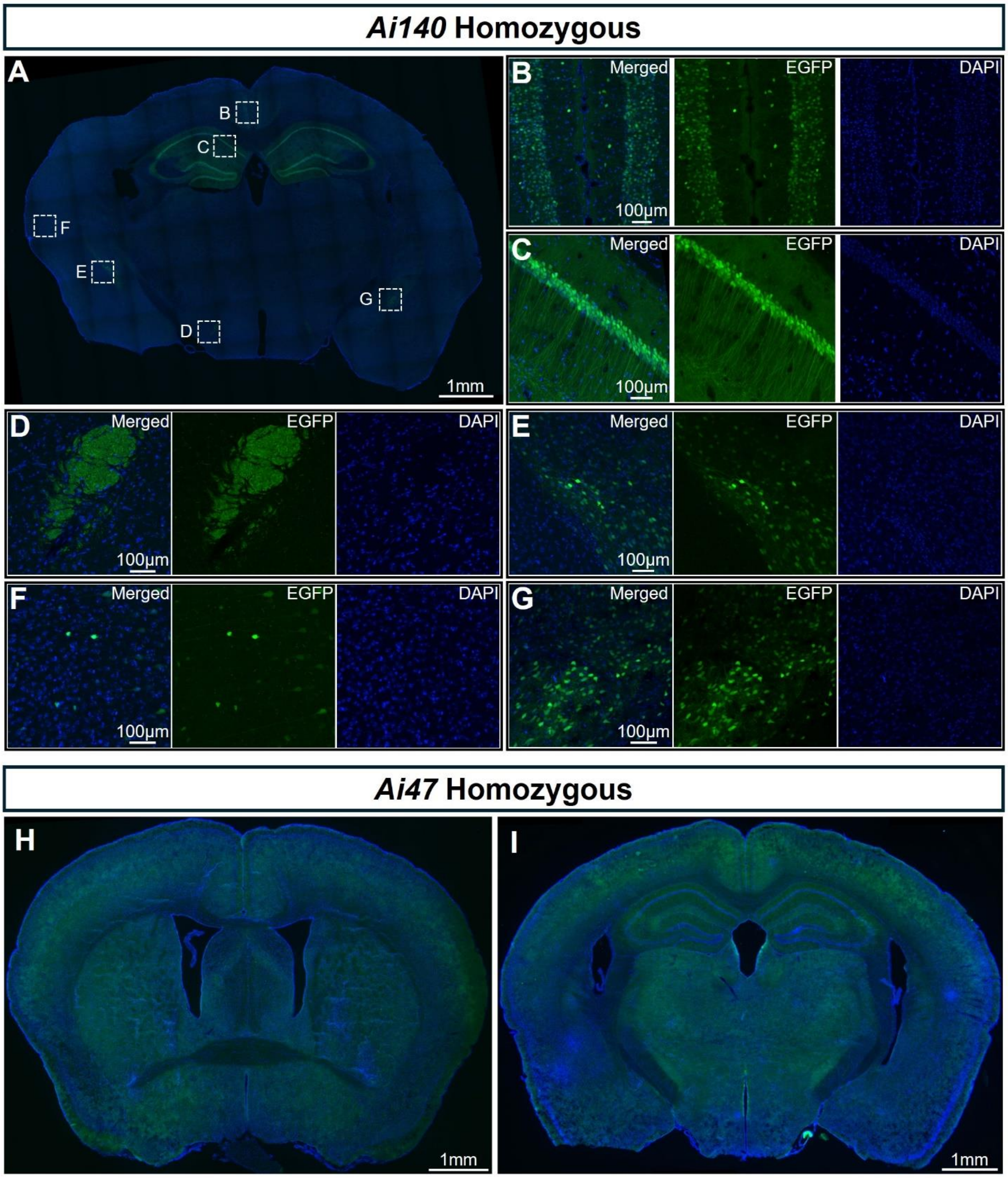

Fig. S1 Examination of non-specific fluorescence leakage in the brains of *Ai140* and *Ai47*. **A** Representative coronal section from an Ai140 mouse at 3 months of age. **B**–**G** Magnified views. **H**, **I** Two representative coronal sections from an Ai47 mouse at 6 months of age. There is no non-specific fluorescence leakage in the Ai47 mice.
